# Addressing Decision-Making Capacity in Application of Involuntary Treatment in Latvia: Case Law Analysis

**DOI:** 10.1192/j.eurpsy.2023.1143

**Published:** 2023-07-19

**Authors:** K. Konstantinova, S. Olsena

**Affiliations:** Faculty of Medicine, University of Latvia, Riga, Latvia

## Abstract

**Introduction:**

A well-established principle is that informed consent is an obligatory requirement for any medical intervention; a patient’s decision-making capacity to consent is a requirement for legally valid consent. Some individuals may be unable to give valid informed consent due to their limited mental capacity. In such cases, laws permit substitute decision-making and involvement of the patient as far as possible (Art.6, Oviedo Convention). National laws of European countries allow persons with mental health problems to be deprived of their liberty and undergo involuntary treatment, namely treatment without a patient’s informed consent, in certain circumstances. Procedural safeguards must be secured, and a court must review its lawfulness (FRA, 2012). The legality of involuntary treatment is highly debated by various audiences (CRPD committee, CoE bodies). In Latvia and other countries, the requirement to assess a person’s decision-making capacity in the application of involuntary treatment is not required.

**Objectives:**

This study was conducted to reveal the role of a person’s decision-making capacity to consent to the treatment of mental disorders in cases where involuntary treatment was approved by courts.

**Methods:**

A retrospective case law study method was applied. Anonymised decisions of Latvian courts at www.manas.tiesas.lv in cases of involuntary treatment in Latvian adult psychiatric hospitals since 2010 were collected and analysed. The content of decisions concerning persons’ decision-making capacity and applicable legal regulations were studied.

**Results:**

The case law revealed that the decision-making capacity had not been addressed regularly and in detail. Latvian law does not require an assessment of capacity, and as a result, the courts do also not require any data. Some elements of decision-making abilities, such as the limited ability to comprehend or process information, are mentioned in the decisions of courts.

**Conclusions:**

There is a need to address the significance of decision-making capacity in the application of patients’ rights law in clinical and legal settings when involuntary treatment is suggested or applied. There is a need to amend the laws justifying the limitations of patients’ rights, particularly concerning involuntary treatment.

**Acknowledgements:**

This paper has been prepared within the research project “Towards a human rights approach for mental health patients with a limited capacity: A legal, ethical and clinical perspective”, No. lzp-2020/1-0397 and the project “Strengthening of the capacity of doctoral studies at the University of Latvia within the framework of the new doctoral model, identification No.8.2.2.0/20/I/006”

**Disclosure of Interest:**

None Declared

